# Immunogenicity of Potomac horse fever vaccine when simultaneously co‐administered with rabies vaccine in a multivalent vaccine or as two monovalent vaccines at separate sites

**DOI:** 10.1111/evj.13096

**Published:** 2019-04-05

**Authors:** H. C. McKenzie, R. A. Funk, L. Trager, S. R. Werre, M. Crisman

**Affiliations:** ^1^ Department of Large Animal Clinical Sciences Virginia Maryland College of Veterinary Medicine Virginia Tech Blacksburg Virginia USA; ^2^ Laboratory for Study Design and Statistical Analysis Department of Biomedical Sciences and Pathobiology Virginia Maryland College of Veterinary Medicine Virginia Tech Blacksburg Virginia USA

**Keywords:** horse, Potomac horse fever, *Neorickettsia risticii*, equine neorickettsiosis, multivalent vaccine, monovalent vaccine

## Abstract

**Background:**

Potomac horse fever (PHF) is a potentially fatal enterocolitis of horses caused by *Neorickettsia risticii*. The disease was originally recognised almost 40 years ago in the state of Maryland in the US. It is now known to occur in many areas of North America, as well as having been described in South America and Europe. Monocomponent PHF vaccines are available, but clinical protection with vaccination has been reported to be inconsistent.

**Objectives:**

This study was designed to assess the immunogenicity of a commercially available Potomac Horse Fever (PHF) vaccine when administered as either a monovalent PHF vaccine simultaneously co‐administered with a separate monovalent Rabies vaccine or as a multivalent PHF/Rabies vaccine in horses.

**Study design:**

Randomised parallel group trial.

**Methods:**

Ninety‐one client or University owned horses participated in this open‐label randomised study, with 45 horses receiving the monovalent vaccines at separate sites and 46 receiving the multivalent vaccine at a single site. Serum PHF IFA titres were determined twice prior to vaccination and at 1, 2 and 3 months after vaccination.

**Results:**

Both vaccination protocols exhibited poor immunogenicity, with only one‐third of all the animals demonstrating seroconversion, defined as an increase in titre of greater than 400 over baseline, at any time point after vaccination. The monovalent PHF vaccine exhibited significantly greater immunogenicity in terms of the number of horses exhibiting seroconversion, as compared to the multivalent vaccine, at one (20 vs. 11, P = 0.03) and two (18 vs. 9, p = 0.02) months post vaccination. The monovalent PHF vaccine also exhibited significantly greater immunogenicity in terms of the median (interquartile range) IFA titres, as compared to the multivalent vaccine, at one (800 [200–1600] vs. 400 [200–800], P = 0.009) and 2 months (400 [200–1600] vs. 400 [100–800], P = 0.02) post vaccination. There was no significant difference between groups at 3 months in either seroconversion rate or median IFA titers.

**Main limitations:**

This study did not assess the actual protective effects of PHF vaccination but rather used the serologic response to vaccination as a surrogate biomarker of immunity.

**Conclusions:**

The multivalent PHF/Rabies vaccine exhibited lower immunogenicity as compared to the monovalent PHF vaccine co‐administered with a separate Rabies vaccine.

## Introduction

Potomac horse fever (PHF), also termed equine neorickettsiosis, is a potentially fatal disease of horses originally recognised in 1979 near the Potomac River in Maryland. It is now known to occur in many areas of North America, as well as having been described in South America and Europe 1. The etiologic agent, originally termed *Ehrlichia risticii*, has been reclassified as *Neorickettsia risticii* 2, 3, 4, 5. *N. risticii* is an obligate intracellular parasite that infects monocytes and macrophages, as well as intestinal epithelial and mast cells 6. The clinical signs typically associated with PHF include diarrhoea, fever, depression, lethargy, anorexia, colic, dehydration and laminitis 7, 8, 9, 10. Only 60–66% of affected horses are reported to develop diarrhoea 7, 11. In some cases of PHF, a fatal outcome may be primarily due to the development of laminitis, which has been reported to occur in up to 36% of cases, even without the development of other clinical signs 7, 12.

While four inactivated vaccines have been licensed for use in horses in the United States only one is currently available, either as a single agent vaccine^1^, ^2^, ^3^, ^4^ or as a multivalent combination with Rabies vaccine^5^ (PHF‐Vax), (PotomacGuard), (PHF‐Gard), (Equine Potomavac) 7. All PHF vaccines have been monocomponent vaccines, containing only one strain of the bacterium, but multiple strains have been identified from horses with natural infection 13, 14. PHF vaccines typically produce weak immune responses which may only lessen the severity of the disease rather than prevent it 13. Vaccine failures are well described, at up to 89% and have been associated with poor antibody production secondary to vaccination 13, 15. Further influences contributing to vaccine failures likely include the antigenic and genomic heterogeneity among *N. risticii* isolates 13, 14, 16, 17, 18.

There are limited studies in the literature describing the effectiveness of the PHF vaccine in increasing serum titers of antibodies to *N. risticii* antigens 13, 19, 20. These studies reported results from small numbers of horses, with high variability in titer responses and only the single agent (monovalent) form of the vaccines were used. No studies have reported the immunogenicity of PHF vaccination when using a multivalent Potomac Horse Fever‐Rabies vaccine. The objective of this study was to determine if the administration of a monovalent PHF vaccine concurrently with a monovalent Rabies vaccine in different sites provides a different serological response when compared to administration of a multivalent Potomac Horse Fever‐Rabies vaccine.

## Materials and methods

This study was reviewed and approved by the Virginia Tech Institutional Animal Care and Use Committee (Protocol #17‐213) and the Virginia Maryland College of Veterinary Medicine Veterinary Teaching Hospital Board. The study was performed during the time period from late November 2017 through March 2018.

### Study subjects

Clients of the Virginia Maryland Veterinary Teaching Hospital Equine Field Service were solicited for potential inclusion of their horses and horses owned by Virginia Tech were also eligible. Ninety‐eight horses were considered for inclusion based upon the following criteria. Horses were to be between 4 and 24 years of age and of any sex. They could have no history of recent illness and they had to have been previously vaccinated for PHF within the last 12 months, but not within the last 3 months. Forty‐six clients and fifty‐two University owned horses were considered eligible for inclusion. These horses underwent a physical examination and were found to have no signs of active clinical illness.

### Randomisation

Horses enrolled into the study were randomised by barn, age and sex using random numbers generated within an Excel^6^ spreadsheet and allocated to either receive a monovalent PHF vaccine co‐administered with a monovalent Rabies vaccine (Monovalent group), or a multivalent rabies/PHF vaccine (Multivalent group).

### Vaccination

The vaccines utilised in this study were: monovalent Potomac Horse Fever vaccine (Equine Potomavac^®^, lot 50089A)^4^, monovalent Rabies vaccine (Imrab^®^, lot 14083) (Imrab^®^)^7^, and a combination Potomac Horse Fever and Rabies vaccine (Equine Potomavac^®^ + Imrab^®^, lot 51071) (Equine Potomavac^®^ + Imrab^®^)^7^. Each horse simultaneously received a single treatment with either co‐administered monovalent PHF and rabies vaccines in two separate sites four inches apart in the left cervical region or the multivalent PHF and rabies vaccine in one site in the left cervical region. All vaccines were administered intramuscularly using a 20‐gauge 1.5‐inch (3.8 cm) needle.

### Immunogenicity assessment

Serum samples were obtained from all eligible horses 1 month prior to the start of the study for screening PHF immunofluorescent immunoassay (IFA) titers. All enrolled horses then had baseline serum samples drawn for PHF IFA titers immediately prior to vaccination. Additional serum samples were subsequently collected at 4, 8 and 12 weeks post vaccination for determination of PHF IFA titers. Sera were separated and stored at −70°C until shipment to the Cornell University Animal Health Diagnostic Center for PHF IFA titer determination. In order to minimise the possibility of inter‐assay variability the screening samples were run as a single batch, prior to the beginning of the trial. All samples from the trial itself (baseline, 4, 8 and 12 weeks) were then run as a single batch following the conclusion of sample collection. PHF IFA titers were performed using the following protocol. Serial dilutions of equine sera from 1:100 to 1:6400 were prepared in Phosphate Buffered Saline (PBS) supplemented with 40% chicken serum, and 100 μL volumes were applied to wells of Teflon‐coated slides containing air dried and acetone fixed with DKDEA4 (Transformed Deer Kidney Cells)^8^ infected with *N. risticii*. Uninfected cells were inoculated with the same serial dilutions. The slides were incubated at 37°C in a humidified chamber for 30 min and quickly rinsed with PBS. This was followed by two 10–15 min rinses in PBS, a final quick rinse of distilled water and then air dried. All wells were stained with fluorescent goat anti‐horse IgG (Alexa‐Fluor^®^ 488‐conjugated AffiniPure Goat Anti‐Horse IgG (H&L))^9^, incubated and rinsed as described above. Cells were counter‐stained in Evan's blue solution (1:10,000) (Evan's Blue cat E2129)^j^ for 8–10 min with a final rinse in distilled water. Slides were read on a Nikon fluorescent microscope^k^, comparing fluorescent reactions of infected vs. non‐infected cells at each dilution to differentiate specific fluorescence from non‐specific background staining. A positive and negative control serum was run concurrently with each set of stained slides.

### Sample size determination and data analysis

A power analysis was performed using data from an earlier study that used the same monovalent PHF vaccine utilised in the current study 13. The analysis was based upon an expectation that the monovalent PHF vaccine would result in an increased serum titer in 80% of the group, with the multivalent vaccine only increasing the titre in 50% of that group. Utilising a target power value of 0.8 the analysis indicated that a minimum of 40 horses would be required in each group.

The statistical analysis was performed with the primary outcomes being serum PHF IFA titers and seroconversion. Seroconversion was defined as an increase of 400 or greater in titer from baseline (time point 1). Besides baseline, data were collected at time points 2 (4 weeks), 3 (8 weeks) and 4 (12 weeks) after vaccination. Normal probability plots showed that titres were skewed. Accordingly, titres were summarised as medians (interquartile range) while seroconversion was summarised as counts and percentages. Titre levels were compared between the two vaccine groups (separately at each time point) using Friedman's Chi‐square. The proportions of horses that seroconverted at time points 1, 2 and 3 were compared between the treatment groups using the Mantel‐Haenszel Chi‐square. Data for time points 2 and 3 were also pooled to constitute time point 2–3 for comparison to time point 1. Both sets of analyses controlled for strata created by matching over barn, sex and age group at the design stage. Statistical significance was set to P<0.05. All analyses were performed using SAS version 9.4.^l^

## Results

### Screening titers

One horse was identified with a PHF IFA titer above 1:800 on the initial screening titre. This horse was eliminated from the study, as an IFA titer of >1:800 is considered to be consistent with recent exposure, recent vaccination or active infection. Ninety‐seven horses were retained in the study.

### Baseline IFA titers

When the results of the baseline titres obtained immediately prior to vaccination were analysed, six horses were identified with PHF IFA titers above 1:800, despite having had titres of <1:800 on their initial screening titers obtained 1 month earlier. These horses were eliminated from the study, as their rising titres prior to vaccination were consistent with active exposure to *N. risticii*. Ninety‐one horses were retained in the final analysis.

### Demographics and baseline characteristics

The multivalent group contained 26 mares and 20 geldings, while the monovalent group contained 21 mares, 21 geldings and 2 stallions. The median age of the horses in the multivalent group was 14 years (s.d. 3.8 years, range 5–20 years), while the median age of the monovalent group was 14 years (s.d. 5.1 years, range 5–23 years).

### Immunogenicity

When considering all horses involved in the study, PHF vaccination demonstrated very inconsistent immunogenicity. At 1‐month post vaccination only 31 of 91 (34%) horses showed seroconversion, defined as an increase in PHF IFA titer of >400 over baseline. This decreased slightly to 27 of 88 (31%) horses at 2 months post vaccination and 24 of 90 (27%) horses at 3 months post vaccination. When comparing the two different vaccination groups there was a significantly greater number of horses demonstrating seroconversion in the monovalent group as compared to the multivalent group at 1‐month post vaccination (20 vs. 11, P = 0.03) and 2 months post vaccination (18 vs. 9, P = 0.02), but no significant difference at 3 months (14 vs. 10, P = 0.2) (Table 1). This equates to 44% of horses demonstrating seroconversion in the monovalent group at 1‐month post vaccination, as compared to only 24% of the horses in the multivalent group, while at 3 months post vaccination 31% of horses demonstrated seroconversion in the monovalent group as compared to 22% of horses in the multivalent group.

**Table 1:** Number of horses exhibiting seroconversion (an increase in PHF IFA titer of >400 over baseline) at each time point

Month post vaccination	Vaccine	n	Seroconverted	Not seroconverted	P value
1	Multivalent	46	11	35	0.03
	Monovalent	45	20	25	
2	Multivalent	44	9	35	0.02
	Monovalent	44	18	26	
3	Multivalent	46	10	36	0.2
	Monovalent	44	14	30	

When considering the median PHF IFA titers between vaccination groups in all horses, there was no significant difference between groups at baseline, but there was a significantly greater median (IQR) IFA titer in the monovalent group as compared to the multivalent group at both 1‐month (800 [200–1600] vs. 400 [200–800], P = 0.009) and 2 months (400 [200–1600] vs. 400 [100–800], P = 0.02) post vaccination. There was no significant difference in median IFA titres between groups at 3 months post vaccination (Table 2). Further evaluation of the IFA titers in the horses that seroconverted supported the relatively greater serologic response to the monovalent vaccination protocol, as the median (IQR) titer in the monovalent group was significantly greater than in the multivalent group at 1‐month post vaccination (1600 [1600–3200] vs. 800 [800–1600], P = 0.002). There was a trend towards a significant difference at 2 months post vaccination (1600 [800–3200] vs. 800 [800–1600], P = 0.05), but there was no significant difference in median titers at 3 months post vaccination (Table 3).

**Table 2:** Summary statistics for PHF IFA titers for all samples

Sample	Vaccine	n	Median (Interquartile range)	P value
Baseline	Multivalent	46	200.0 (100.0–400.0)	0.9
	Monovalent	45	100.0 (100.0–400.0)	
1 month	Multivalent	46	400.0 (200.0–800.0)	0.009
	Monovalent	45	800.0 (200.0–1600.0)	
2 months	Multivalent	44	400.0 (100.0–800.0)	0.02
	Monovalent	44	400.0 (200.0–1600.0)	
3 months	Multivalent	46	400.0 (100.0–800.0)	0.3
	Monovalent	44	400.0 (100.0–800.0)	

**Table 3:** Serum IFA titers of horses that seroconverted (an increase in PHF IFA titer of >400 over baseline) following vaccination

Month post vaccination	Vaccine	n	Median (Interquartile range)	P value
1	Multivalent	11	800.0 (800.0–1600.0)	0.002
	Monovalent	20	1600.0 (1600.0–3200.0)	
2	Multivalent	9	800.0 (800.0–1600.0)	0.05
	Monovalent	18	1600.0 (800.0–3200.0)	
3	Multivalent	10	1200.0 (800.0–1600.0)	0.7
	Monovalent	14	1600.0 (800.0–1600.0)	

In order to address the possible influence of repeated assessments the data were also analysed using a pooled approach, wherein the results from the two time points where significant differences in vaccine responses had been observed (1 and 2 months post vaccination) were grouped together. Using this approach, a horse was considered to have seroconverted if it had an increase in PHF IFA titer of >400 at either 1 or 2 months after vaccination. This analysis revealed that within this time period 21 horses (46.7%) in the monovalent group seroconverted, while only 12 horses (26.15%) in the multivalent group seroconverted (P = 0.02). When the median IFA titers from the two treatment groups were compared using this approach, there was a significant difference, with the monovalent group having median titers of 800 (200–1600) as compared to the multivalent group which had median titers of 400 (200–800) (P = 0.005).

## Discussion

A monovalent inactivated whole cell PHF vaccine^4^ is commercially available based on a 1984 isolate of *N. risticii* 19. Over the ensuing 30 years, multiple strains of *N. risticii* have been isolated from horses with PHF demonstrating significant genetic and antigenic strain variation 14, 16, 18, 20, 21. Additionally, vaccine efficacy has been reported to be marginal, likely due to deficient immunogenic responses 13, 20, 22. Studies have suggested that strain variants having pathogenic, immunologic and molecular differences may all contribute to vaccine failure 13, 23. Large controlled field studies evaluating the antibody response of horses to PHF vaccination have not been reported. This was a prospective, single‐blind, randomised, parallel‐group clinical study designed to evaluate the immunogenicity of PHF vaccine given either simultaneously in separate injections with a rabies vaccine, or as a combined PHF‐rabies vaccine. To our knowledge, this is the largest study evaluating PHF vaccine responses in horses. Overall, PHF vaccination demonstrated very limited immunogenicity regardless of group. At 1‐month post vaccination only 34% of horses showed seroconversion and this decreased slightly to 31% of horses at 2 months post vaccination and 27% of horses at 3 months post vaccination. However, the greatest differences were observed between the monovalent group and the multivalent group, with significantly fewer horses in the multivalent group exhibiting seroconversion at 1 and 2 months post vaccination. At 3 months there was no statistical difference in the groups. These data suggest that in addition to the overall poor immunogenicity of the vaccine, the rabies fraction in the multivalent vaccine may have interfered with the antibody response to the PHF fraction of the vaccine. Alternatively, some difference in the formulation of the vaccines, such as the PHF antigen load, may be responsible for the reduction in immunogenicity of the multivalent vaccine.

All horses enrolled in the study were participants in a preventative medical program and vaccinated routinely, including PHF vaccination. Horses had been vaccinated once or twice a year depending on potential exposure and owner's preference. Previously vaccinated horses were used in this study as this was likely more representative of vaccine performance in real world settings where horses in endemic areas are likely to have been previously vaccinated or potentially previously exposed to *N. risticii*. The use of previously vaccinated horses should also have provided for more consistent and measurable increases in IFA titers, as vaccination was eliciting a memory response rather than a priming response. The study was performed during the winter months in order to decrease the likelihood of active exposure to *N. risticii* during the sampling period. The fact that six horses had to be eliminated from the study due to rising titers between the time of the screening titers in November and the baseline titers in December indicated that natural exposure had occurred at some time in November or early December. This may have been due to warmer than normal temperatures and higher than normal rainfall during November 2017 as compared to annual averages (https://www.weather.gov/rnk/climatePlotsBcb). Despite these apparent exposures to *N. risticii*, none of the horses in the study exhibited clinical signs of disease during the course of the study. It is also possible that natural exposure could have occurred during the months of December through March and that this could have accounted for some of the increases in IFA titers noted during the study. This seems unlikely as those months are the coldest of the year in the study location, and the historical weather data indicate that December through February were actually colder than historical averages (https://www.weather.gov/rnk/climatePlotsBcb).

This study demonstrated a significant difference in immunogenicity between the two vaccination protocols for PHF vaccination, with the use of a monovalent PHF vaccine administered separately from a monovalent rabies vaccine yielding a more pronounced immunologic response than the multivalent PHF/rabies vaccine. This outcome is of interest as the PHF vaccine was already established to have inconsistent immunogenicity and incomplete protection from disease, therefore any further reduction in immunogenicity may render the vaccine even less effective in the field setting. While it has been established that the serologic response to PHF vaccination is not well correlated with disease prevention, the low immunogenicity of this vaccine likely contributes to the well described low level of protection from disease. Indeed, the primary benefits attributed to PHF vaccination are decreasing the severity of clinical signs and lessening the development of serious sequalae, such as laminitis 19.

Multivalent vaccines should ideally generate a protective immune response to each of their constituents that is equivalent to that achieved when they are administered as monovalent vaccines. Unfortunately, there are situations where combining antigens in a vaccine formulation results in reduced immunogenicity and/or protection from disease 24, 25, 26, 27, 28. There are several potential causes for reduced immunogenicity of multivalent vaccines, which include: physical or chemical interactions including incompatibility of antigens or interference of additives (e.g. adjuvant, preservative), immunological interference that may arise from some antigens sharing common epitopes, pre‐existing immunity against one vaccine component and viral competition within or between live‐attenuated vaccines 29, 30. It is also possible that the PHF antigenic load differs between the monovalent PHF vaccine and the multivalent PHF‐Rabies vaccine, but that data is not available from the manufacturer. It is unclear what is responsible for the observed reduction in the immunogenicity of PHF vaccine when combined with Rabies vaccine as documented in this study.

### Conclusion

The monocomponent PHF vaccine examined in this study exhibited poor immunogenicity overall, consistent with previous reports. The immunologic response to this vaccine was weaker when it was administered as a multivalent PHF/Rabies vaccine, which has not been previously reported. The mechanism responsible for this phenomenon was not investigated in this study. These results suggest that the PHF vaccine should be administered as a monovalent vaccine in order to maximise the immunologic response to vaccination. While this study did not assess the actual protective effects of PHF vaccination the poor immunogenicity observed in both treatment groups reinforces that efforts to develop a more immunogenic and protective vaccine could improve the health and welfare of horses in areas with endemic PHF.

## Authors’ declaration of interests

M. Crisman is an employee of Zoetis, Inc. The remaining authors declare no competing interests.

## Ethical animal research

This study was reviewed and approved by the Virginia Tech Institutional Animal Care and Use Committee (Protocol #17‐213) and the Virginia Maryland College of Veterinary Medicine Veterinary Teaching Hospital Board.

## Owner informed consent

Owners gave consent for their animals’ inclusion in the study.

## Source of funding

This project was supported by Zoetis, Inc.

## Authorship

H. McKenzie and M. Crisman conceived and designed the study, with the assistance of S. Werre. R. Funk, L. Trager and H. McKenzie performed the study. H. McKenzie, M. Crisman, R. Funk and S. Werre were involved in the analysis or interpretation of the data. All authors contributed to the writing/reviewing of the paper and approved the final version for submission.

## Manufacturers' addresses

1 Schering Plough/Merck, Whitehouse Station, New Jersey, USA: 1987 to 2008.

2 Fort Dodge/Pfizer, Madison, New Jersey, USA: 1993 to 2011.

3 Pfizer Inc., Madison, New Jersey, USA: 2000 to 2001.

4 Merial Inc., Duluth, Georgia, USA: currently available.

5 Merial Inc., Duluth, Georgia, USA.

6 Microsoft Corporation, Redmond, Washington, USA.

7 Merial Inc., Duluth, Georgia, USA.

8 AHDC, Cornell University, Ithaca, New York, USA.

9 Jackson Immunoresearch, West Grove, Pennsylvania, USA.

10 Sigma, St Louis, Missouri, USA.

11 Nikon, Shinagawa, Tokyo, Japan.

12 SAS Institute Inc., Cary, North Carolina, USA.
